# Nanomaterials for Removal and Speciation of Chromium

**DOI:** 10.3390/ma18071485

**Published:** 2025-03-26

**Authors:** Krystyna Pyrzynska

**Affiliations:** Faculty of Chemistry, University of Warsaw, 02-093 Warsaw, Poland; kryspyrz@chem.uw.edu.pl

**Keywords:** nanomaterials, chromium, adsorption, removal, speciation

## Abstract

The removal of chromium compounds, particularly its more toxic Cr(VI) form, from industrial wastewater is important as it causes serious environmental and health issues. Adsorption processes have attracted continuous interest for solving these problems due to the diverse range of various adsorbents. Nanomaterials are increasingly employed as novel sorbents as they have a large specific surface area and high chemical stability. Functionalization of their surface by covalent or noncovalent interactions with other components, grafting or doping with heteroatoms can enhance separation and removal efficiency. This paper aims to provide insights into the recent progress in the application of nanomaterials for chromium removal from aqueous solutions and speciation analysis of it.

## 1. Introduction

Most environmental chromium originates from anthropogenic sources as it is widely used in several industries such as metallurgy, plating, leather tanning, and paint production. Geogenic processes also contribute to the chromium levels in groundwater, though to a lesser extent. Chromium primarily exists in aqueous environments as Cr(III) and Cr(VI) species [1]. The biological and toxicological properties of chromium depend on its oxidation state. While Cr(III) is an essential trace element required for normal glucose, protein, and lipid metabolism, used in nutritional supplementation [2], Cr(VI) is highly toxic, and causes oxidative stress, DNA damage, and epigenetic gene modifications [3]. Recent studies on Cr(III) essentiality and Cr(VI) toxicity have been extensively reviewed [4,5].

Chromium speciation in different environmental samples usually involves distinguishing Cr(III) from Cr(VI). The term “total chromium” often refers to the sum of all Cr forms, but in practice, it primarily comprises these two species and comes down to determining the sum of those two main species. Regulations and directives governing chromium emissions in aquatic environments have been reviewed in the literature [6]. According to the World Health Organization, the maximum permissible concentrations of Cr(VI) and total chromium in drinking water are 20 µg/L and 50 µg/L, respectively [7].

The determination of chromium and its speciation has become significant for monitoring water pollution and assessing its impact on human health. Chromatographic techniques coupled with molecular and elemental sensitive detectors are commonly used for the identification and determination of chromium species [8,9,10]. Although inductively coupled plasma mass spectrometry (ICP MS) offers a low detection limit, matrix interferences and species interconversion can pose challenges [10]. Consequently, matrix removal is often necessary. Sorptive extraction techniques allow for the simultaneous separation and preconcentration of one or both chromium species before analysis. Solid phase extraction (SPE), dispersive micro solid phase extraction (DMSPE), and solid phase microextraction (SPME) are widely employed for metal ion separation and preconcentration [11,12,13]. Additionally, Fe_3_O_4_-based sorbents enable easy separation via external magnetic forces, eliminating the need for filtration or high-speed centrifugation [14,15].

A variety of solid materials have been explored for the removal of chromium ions from contaminated waters [16,17,18,19,20,21,22,23]. Among novel sorbents, nanomaterials have gained attraction in analytical applications involving diverse extraction techniques [24,25,26,27]. With sizes ranging from 1 to 100 nm, nanomaterials exhibit high surface areas and mechanical stability. Surface functionalization through covalent or noncovalent interactions, grafting, or heteroatom doping enhances separation and preconcentration efficiency [28,29,30,31,32]. Sorption efficiency depends on pore size, surface area, morphology, and functional groups. Additionally, factors such as sample pH, sorbent dosage, analyte concentration, extraction time, and temperature influence performance.

This paper presents an overview of recent advancements in the application of nanomaterials for chromium removal and speciation analysis from aqueous solutions published in the last five years. Previous contributions are available in review papers [15,18,20,21,33]. Recent reviews predominantly discuss specific adsorbents, such as polymeric materials [34], graphene and carbon nanotubes [35], metal–organic frameworks [36], and layered double hydroxides (LDH) [37]. Other reviews only address the removal of chromium from contaminated waters [38] or magnetic nanostructure applications [15]. This study provides a timely update on recent research findings in the field.

## 2. Removal of Chromium by Nanomaterials

As chromium is extensively used in various industries, wastewater discharge leads to its accumulation in the environment, posing a significant threat. Various strategies have been used for the removal of heavy metals from water and wastewater, such as chemical precipitation using hydroxides or sulfides, ion exchange, flotation, evaporation, membrane filtration, reverse osmosis, coagulation, and electrochemical separation. New methods include membrane bioreactors, forward osmosis, and electrocoagulation. Each method for removing toxic Cr(VI) forms has some advantages and drawbacks. All these methods were thoroughly presented and discussed in recent reviews [16,39]. Among these methods, adsorption using batch and column methodologies remains a simple, eco-friendly, and regenerable approach that is easy to design [39,40]. Cr(VI) is highly soluble, bioavailable, and toxic, as opposed to Cr(III) species, making its removal a priority [40].

**Figure 1:**
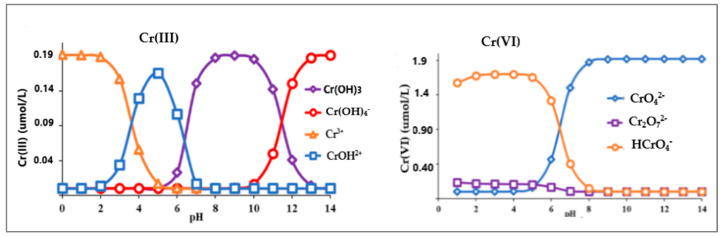
Speciation profile of chromium in aqueous solution at different pHs. Reproduced under the terms of a CC BY license from reference [41]. Copyright 2019 Elsevier.

Nanomaterials’ properties, such as surface area, charge, pore diameter, and functional groups, influence adsorption efficiency. In addition, sample pH affects sorption yields by altering surface protonation chromium speciation. The speciation profile of chromium in aqueous solutions at different pH is depicted in Figure 1 [41]. Under acidic conditions, Cr(III) forms cationic hydroxo- and aqua complexes, whereas in neutral and weakly alkaline solutions, it precipitates as Cr(OH)_3_. In alkaline conditions, negatively charged Cr(OH)_4_^−^ complexes dominate. Cr(VI) exists as HCrO_4_^=^ and Cr_2_O_7_^2−^ in acidic conditions, transitioning to predominantly CrO_4_^2=^ at higher pH values. in contrast, as the pH of a solution increases, more groups on the adsorbent surface groups are ionized and the surface charge becomes more negative.

The adsorption process is more efficient when the sample’s pH is higher than the nanoparticle’s point of zero charge due to the electrostatic interaction. Adsorption efficiency generally increases with higher adsorbent dosage and longer extraction time until equilibrium is reached. However, the competitive effects of other matrix components, such as cations for Cr(III) sorption and anions for Cr(VI), can reduce efficiency. A high sorbent capacity and selectivity are therefore desirable attributes.

Adsorption isotherm and kinetics models can provide information about the adsorption process, maximum adsorption capacity (q_max_), and mass transfer steps, which is essential to evaluate the performance of a given adsorbent [42,43]. Adsorption capacity is measured at an equilibrium state (saturation), so it is independent of the amount of adsorbent and reflects the ability of adsorbents to take up that particular adsorbate. Thus, its value could be used for comparisons with other materials. Removal efficiency is another operative parameter used quite often in the data in the literature to assess the sorption properties of nanomaterials [42,44]. However, its value depends on the sorbent dosage and the initial concentration of a given species. Studies on adsorption isotherms using various models provide insight into adsorption processes (monolayer/multilayer adsorption) and the maximum adsorption capacity (q_max_), which is essential for system design [42]. Monolayer or multilayer adsorption can be distinguished by modeling the experimental adsorption data with the relevant adsorption models (e.g., Langmuir, Freundlich, Temkin, Sips, and D-R isotherms).

The mass transfer from chromium ions to adsorbents includes external/internal diffusion and adsorption onto active sites [43]. The study of adsorption kinetics can be used to calculate the adsorption rate and reveal the rate-limiting steps. Understanding the rate-limiting step can help to improve the performance of an adsorbent. For example, if internal diffusion is the rate-limiting step, making the adsorbent more porous can speed up the adsorption process [44].

The temperature in an adsorption process is another factor influencing the adsorption efficiency. Most of Cr adsorption processes are spontaneous and endothermic reactions. Consequently, the adsorption capacity increases as temperature rises, typically for chemisorption.

The main group of nanomaterials used for the enrichment and removal of chromium is presented below.

### 2.1. Carbon Nanotubes

Dehghani et al. used primary multi-walled carbon nanotubes and single-walled carbon nanotubes for Cr(VI) removal with a sorption capacity of 1.26 and 2.35 mg/g, respectively [45]. The maximum efficiency was noted at pH 2.5, where the oxo-anionic form of Cr (VI) is predominant and ion exchange and electrostatic interactions take place between -OH and -COOH functional groups on the CNT’s surface. Cr(VI) ion removal was affected by the presence of competing SO_4_^2−^ anions. In the batch study by Mpouras et al., the maximum sorption of Cr(V) onto MWCNTs (~450 mg/kg) was determined for sample pH values up to 6.3 (the value of point zero charge, PZC) and initial chromium concentration of 250 mg/L [46]. For a pH higher than the PZC, the sorption rapidly decreased since the negatively charged surface could not attract Cr(VI) anions. In the case of a lower initial chromium concentration (11 mg/L), the value for Cr(VI) maximum sorption (~10 mg/kg) was obtained at pH values up to 8. According to Huang et al., the mechanism for the removal of hexavalent chromium ions by MWCNTs is attributed to electrostatic attractions and the reduction of adsorbed Cr(VI) to Cr(III) by the surface hydroxyl groups [47]. These processes were intensely promoted by the addition of humic acid and surfactants. Cr(VI) was also removed via chemical reduction under acidic conditions to Cr(III) using H_2_O_2_ released from CNTs modified with nano CaO_2_ [48].

Carbon nanotubes combined with metallic oxides can improve the Cr(VI) adsorption capacity. For example, nano-sized hydrated zirconium oxide ZrO(OH)_2_ particles were deposited onto the surface of CNTs and used to remove Cr(VI) ions [49]. This combination allows for its high sorption reactivity to be used effectively, with several hydroxyl groups playing a crucial role. The sorption reached equilibrium within 6 h, with an adsorption capacity of 97.39 mg/g. The MWCNTs fitted with iron–manganese binary oxide exhibited the maximum adsorption capacity of 47.25 mg/g at pH 2 for Cr(VI) with the main role of electrostatic attraction [50]. It was found that a redox transformation occurred between Cr(VI) and As(III) after these two species were co-sorbed onto the FeMnOx@MWCNT’s surface.

A highly effective approach to enhancing Cr(VI) removal involves nitrogen doping CNTs [51,52,53]. Cheng et al. proposed magnetic N/Fe-doped carbon nanotubes prepared by the pyrolysis of waste plastics in the presence of urea using Fe(NO_3_)_3_ as a catalyst [51]. Hydroxyl-, carboxylic-, and nitrogen-containing groups were involved in Cr(VI) removal. Additionally, Fe^0^-, Fe^2+^-, and H*-reducing radicals generated during iron corrosion in an acidic medium facilitated the reduction in Cr(VI) to Cr(III). The determined sorption capacity was 27.47 mg/g at pH 3. Huang et al. reported an exceptionally high maximum sorption capacity for Cr(VI) (970.87 mg/g), calculated using the Langmuir isotherm model, on nitrogen-doped magnetic carbon nanotubes synthesized with melamine and FeCl_3_ as precursors [52]. The tubular structure with encapsulated ferric carbide was resistant to acid corrosion. The spent sorbent could be reused as a catalyst for the electrochemical reduction of CO_2_.

Carbon nanotube–polymer composites combine the excellent functional properties of CNTs with the favorable processing characteristics of polymers [54]. However, the dispersion and alignment of CNTs in the polymeric present challenges. Consequently, significant efforts are being made to improve the production processes used for such nanocomposites. Mahpishanian et al. synthesized nanocomposites using a high internal phase emulsion templating approach (involving 2-ethylhexyl acrylate and ethylene glycol dimethacrylate monomers), where SWCNTs were embedded within the polymeric matrix pores [55]. This nanocomposite selectively removed Cr(VI) with a maximum sorption capacity of 72.35 mg/g. Polyamine-modified multiwalled carbon nanotubes (PA@MWCNTs) exhibited a fast Cr(VI) sorption rate, achieving 95% removal within 3 min at pH 4 [56]. PA-MWCNTs nanomaterial displayed a maximum Cr(VI) sorption capacity of 168.54 mg/g, benefiting from the introduced surface amine groups.

A novel nanomaterial prepared by modifying oxidized MWCNTs with *Citrus sinensis* juice (OJMW) was proposed by Amaku and Taziwa [57]. Orange juice, which was used as a modifier, contains several biocompounds with reductive potential. The primary Cr(VI) removal mechanism by these carbon nanotubes involves electrostatic interaction between the positively charged surface and anionic chromium species, followed by the reduction of Cr(VI) to Cr(III) and its subsequent adsorption onto the surface and internal pores. The optimal removal of Cr(VI) was achieved at pH 2 with a 3 h contact time. The maximum sorption capacity of these relatively cheap modified MWCNTs was 60.91 mg/g, compared to 44.72 mg/g for oxidized MWNTs alone. The thermodynamic and kinetic studies revealed that Cr(VI) removal using MWCNTs is an exothermic process and follows a pseudo-first-order kinetic model. In contrast, modified nanotubes exhibited an endothermic process and followed a pseudo-second-order kinetic model. After Cr(VI) adsorption, the adsorbent was washed with diluted HCl for regeneration following a recycling process with 0.5 M NaOH. A removal efficiency of over 70% was observed after the fourth adsorption–desorption cycle. However, the authors did not specify the reproducibility of the carbon nanotube modification method.

### 2.2. Graphene Derivatives

Graphene oxide (GO), produced by the oxidation of naturally occurring graphite, exhibits good hydrophilicity and dispersion in water, unlike graphene and graphite, due to the presence of multiple –OH and –COOH groups attached to its layers. GO can easily form magnetic nanocomposites through electrostatic interactions between its negatively charged nanosheets and the positively charged surface of Fe_3_O_4_, simplifying the separation process. Additionally, GO can be functionalized with the appropriate groups to form hybrid nanoparticles [58,59]. Reduced graphene oxide (rGO), obtained from GO by partial deoxygenation, behaves as a hydrophilic–hydrophobic balanced sorbent [60].

**Figure 2:**
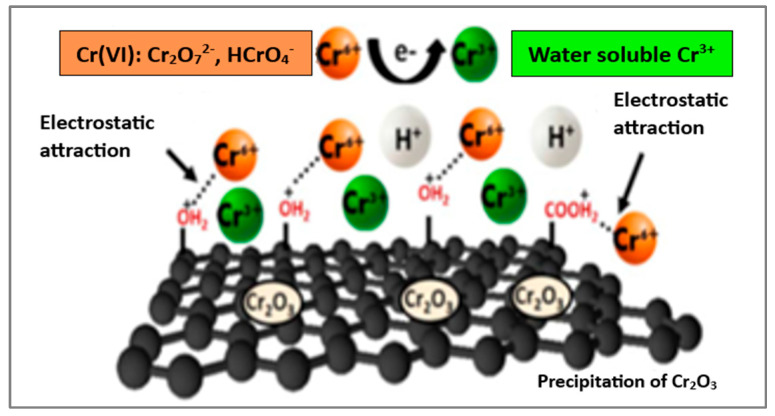
Proposed mechanism for Cr(VI) removal by GO in an acidic environment. Reproduced under the terms of a CC BY license from reference [61]. Copyright 2024 Royal Society of Chemistry.

Cr(VI) sorption on pure GO under acidic conditions primarily involves electrostatic attraction between the protonated oxygen-containing groups on its surface and anionic chromium species in solution, the redox reaction of Cr(VI) to precipitation of Cr(III), and also pore filling (Figure 2) [61]. Reported maximum adsorption capacities for Cr(VI) vary widely, e.g., from 1.222 mg/g [62] to 41.27 mg/g [61]. These differences likely result from the use of different oxidants, additional pre-oxidation treatment, and the application of ultrasound or microwaves to support the reaction.

**Figure 3:**
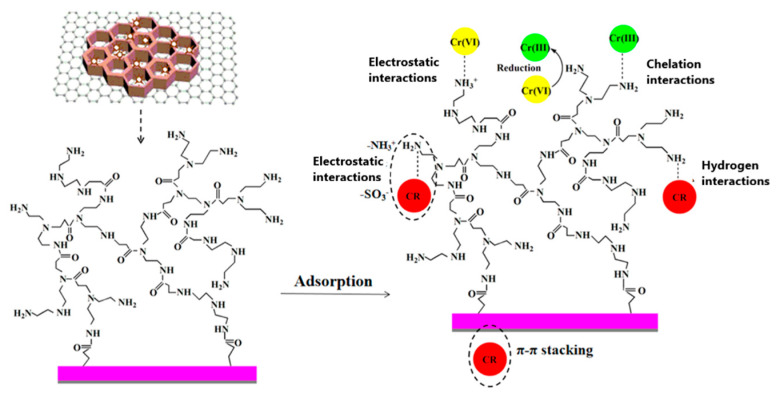
Adsorption mechanism of chromium on GO@SiO_2_ modified with hyperbranched polymer. Reproduced with permission from reference [65]. Copyright 2020 American Chemical Society.

Modifying a GO surface with other materials helps overcome self-aggregation, increases adsorption sites, and enables additional interactions, thereby enhancing removal efficiency. Guo and Bulin reported that under acidic conditions Cr(VI) is adsorbed by magnetic GO@ Fe_3_O_4_ in the form of H_2_CrO_4_, with over three-fifths of H_2_CrO_4_ being reduced to Cr(III), achieving 95.53% adsorption within 10 min [63]. Under basic conditions, chromium is adsorbed as CrO_4_^2−^ without reduction. The reported maximum sorption capacities for GO@ Fe_3_O_4_ under acidic pH were 382.10 mg/g [63] and 140.8 mg/g [64]. Zhang et al. synthesized a graphene oxide/mesoporous silica nanosheet with multistage pores, followed by modification with a hyperbranched polymer containing imino groups for Cr(VI) removal [65]. Using a solution with a 10 mg/L concentration and pH 2, an adsorption efficiency of 87% was achieved with only 5 mg of this nanocomposite. The adsorption process for hexavalent chromium, involving several interactions, is illustrated in Figure 3.

According to Jibin et al., graphene oxide with silica modified with 3-aminopropyltriethoxysilane (APTES) created a synergistic effect in Cr(VI) adsorption at pH 3, reaching a maximum adsorption capacity of 92.28 mg/g [66]. A similar adsorbent, produced by covalently bonding 4-amino-3-hydrazino-5-mercapto-1,2,4-triazole (AHMT) onto GO, demonstrated a high performance not only for hexavalent chromium ions (q_max_ of 734.2 mg/g at pH 2) but also for Hg(II) (q_max_ of 1091.1 mg/g) [67]. The maximum adsorption capacities according to the Langmuir adsorption model for other metal ions on GO-AHMT decreased in the following order: Ag (I) (206.9 mg/g) > Cu(II) (168.9 mg/g) > Pb(II) (103.4 mg/g) > Cd(II) (101.0) > Zn(II) (77.4 mg/g) > Co(II) (43.6 mg/g) > Ni(II) (22.1 mg/g) > Cr(III) (43.6 mg/g). In another study, the deposition of ZnO nanoparticles on graphene oxide improved Cr(VI)’s adsorption kinetics and removal efficiency at a near-neutral pH of 8.02 [68]. The maximum adsorption of Cr(VI) on a GO@zinc molybdenite nanosorbent was obtained using 6 mg of adsorbent at pH 2 with a contact time of 120 min [69].

The novel adsorbent was synthesized by introducing an APTES bridging agent onto the GO surface, which enabled the covalent conjugation of poly(allylamine hydrochloride) (PAH) through Schiff base reactions [70]. The obtained nanosorbent, named PAH-AS@GO, exhibited a high Cr(VI) adsorption capacity of 373.1 mg/g and could effectively decrease its concentration from 9.9 mg/L to below 0.05 mg/L within 50 s with an adsorbent dosage of 0.3 g/L. To facilitate faster collection and reuse, a magnetic version of this nanosorbent was synthesized incorporating Fe_3_O_4_. The maximum experimental adsorption capacity of PAH-AS@GO/Fe_3_O_4_ for Cr(VI) was 219 mg/g. Recently, GO adsorbent functionalized with another Schiff base (4-amino-5-(2-((E)-4-((E)-((3-triethoxysilyl) propyl)imino) methyl) benzylidene) hydrazineyl)-4H-1,2,4-triazole-3-thiol) was proposed for the removal of Cr(VI) from surface runoff, achieving a q_max_ value of 243.3 mg/g [71].

Chitosan (CS), a natural polymer known for its excellent adsorption and coating properties, biocompatibility, and availability, has proven to be effective in removing several environmental pollutants [72]. The amino groups in chitosan can interact electrostatically with anionic analytes and metal ions through adsorption, ion exchange, or chelation. Graphene oxide-modified chitosan nanocomposites exhibited numerous complexing sites and high stability [73,74,75,76,77]. Studies conducted by Matei et al. demonstrate that chitosan follows a monolayer adsorption mechanism for Cr(VI), although with a low capacity of 3.03 mg/g [73]. A significantly higher adsorption capacity (104.16 mg/g) was achieved using a chitosan-grafted graphene oxide nanocomposite (CS@GO) synthesized via ultrasound radiation [74]. The removal efficiency of Cr(VI), however, decreased with increasing concentrations of anions, such as SO_4_^2−^, HPO_4_^2−^, and NO_3_^−^. This nanomaterial can be regenerated using NaOH solution and reused for up to ten cycles. The maximum monolayer adsorption capacity of Cr(VI) reached 100.51 mg/g with a CS@Fe_3_O_4_@GO nanocomposite, but in the presence of sulfate ions, this value dropped by approximately 20 mg/g [76].

Kang et al. [78] proposed reduced graphene oxide (rGO) decorated with iron and nickel nanoparticles, synthesized via a reaction with NaBH_4_. This rGO@Ni-Fe nanomaterial exhibited an adsorption capacity of 197.43 mg/g at pH 5. Cr(VI) was adsorbed onto the rGO surface and locally reduced by the embedded nano-iron particles. The addition of a small amount of nickel enhanced the adsorption performance. However, reusability studies revealed that the nanosorbent almost completely lost its Cr(VI) reduction ability after multiple cycles. The strong acidic or basic eluents used for regeneration dissolved the formed Cr(OH)_3_ and degraded other components on the rGO@Fe−Ni surface. A composite of rGO with ZnO, synthesized from sucrose and zinc acetate via a simple pyrolysis method, Cr(VI) showed an adsorption capacity of 13.5 mg/g—five times higher than that of pure rGO [79]. Notably, its adsorption efficiency was improved under light irradiation due to photocatalytic effects.

### 2.3. Layered Double Hydroxides

**Figure 4:**
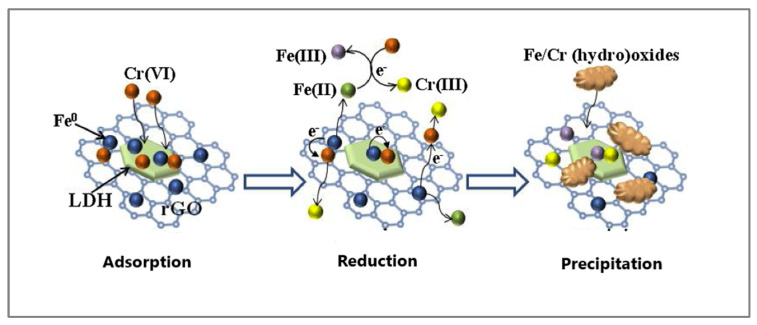
Schematic description of Cr(VI) removal by rCO-Fe-LDH. Reproduced with permission from reference [83]. Copyright 2019 Elsevier.

Layered double hydroxides (LDHs) are two-dimensional nanostructures composed of positively charged metal oxides with charge-balancing hydrated anions intercalated between them [80]. Several mechanisms have been proposed for the adsorption of metal ions, including isomorphic substitution, surface complexation, precipitation, electrostatic interaction, and chelation. Intercalating GO into LDH structures can enhance both adsorption capacity and selectivity [81,82,83]. For instance, GO@NiFe@LDH demonstrated a Cr(VI) adsorption capacity of 53.6 mg/g at pH 3, outperforming NiFe@LDH microspheres [82]. A ternary rGO-Fe-LDH nanohybrid exhibited a good dispersibility in aqueous solutions, improved hydrophilicity, and a positively charged surface, which facilitated the adsorption of anionic Cr(VI) species [83]. Furthermore, adsorbed Cr(VI) was reduced, and the resulting Cr(III) and Fe(II) co-precipitated as oxides or hydroxides. A schematic representation of Cr(VI) removal by rGO@Fe@LDH is shown in Figure 4 [78].

### 2.4. Molybdenum Disulfide

Molybdenum disulfide (MoS_2_), a 2D layered nanomaterial, is widely used in water treatment due to its large surface area and abundance of sulfur-containing functional groups [79,84,85]. Each monolayer consists of a central molybdenum sheet sandwiched between two sulfur sheets with strong interlayer S-Mo-S bonds and weak interlayer van der Waals forces.

**Figure 5:**
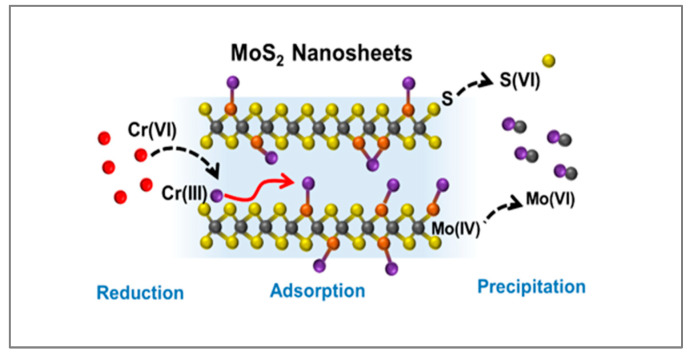
Schematic illustration of removal mechanisms between Cr species and MoS_2_ nanosheets. Reproduced with permission from reference [86]. Copyright 2022 Elsevier.

The 2D flake-like structure of MoS_2_ nanosheets enabled the formation of aligned and ion-accessible nanochannels, where Cr(VI) species are accommodated and reduced. Han et al. observed the complete removal of Cr(VI) at pH 2, with a parallel formation of Cr(III) at an equivalent concentration (~50 mg/L) [86]. This suggests that Cr(VI) reduction is the primary mechanism for its removal by MoS_2_. Additionally, the resulting Cr(III) species can be precipitated with MoO_4_^2−^ and become sequestered within the nanochannels. A schematic representation of Cr species interaction with MoS_2_ nanosheets is provided in Figure 5 [86]. The incorporation of carbonaceous materials prevents the agglomeration of MoS_2_ sheets and introduces additional oxygen groups. A flower-like MoS_2_@GO nanocomposite, synthesized via the hydrothermal method using thioacetamide as a sulfur source, exhibited an adsorption capacity toward Cr(VI) of 80.8 mg/g at pH 2 [87].

### 2.5. Other Nanocomposites

**Figure 6:**
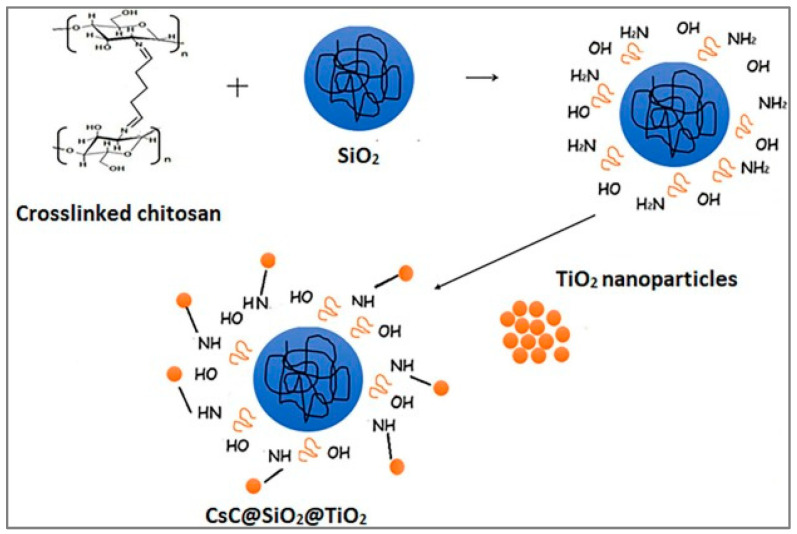
Schematic presentation of CS@SiO_2_@TiO_2_ nanocomposite synthesis. Reprinted under the terms of a CC BY license from reference [89]. Copyright 2023 Japan Oil Chemists’ Society.

A chitosan@TiO_2_ nanocomposite prepared via the photolysis method exhibited a remarkably high adsorption capacity (488 mg/g) for Cr(VI) [88]. The incorporation of SiO_2_ and nanocrystalline TiO_2_ into a chitosan matrix using glutaraldehyde crosslinker was employed for the synthesis of CS@SiO_2_@TiO_2_ nanocomposite (Figure 6) [89]. Under optimum conditions (pH 3.0, contact time of 120 min), the monolayer adsorption capacity reached 182.43 mg/g. CS@SiO_2_@TiO_2_ demonstrated over 90% removal efficiency for Cr(VI) from the solution of 100 mg/L and an adsorbent dose of 50 mg. Notably, the adsorption capacity showed negligible variation after five adsorption–desorption cycles, followed by a NaOH solution regeneration. For a 3D magnetic CoFe_2_O_4_@SiO_2_-NH_2_ nanocomposite, an adsorption capacity of 126.8 mg/g for Cr(VI) has been reported via adsorption and reduction processes [90]. Chloride and nitrate anions had almost no effect on the adsorption capacity values, but other common anions (SO_4_^2−^, PO_4_^3−^) and cations (Mg^2+^, Ca^2+^) had negative effects.

In summary, various synthesized nanomaterials have been proposed for the sorptive removal of Cr(VI) ions. Table 1 presents their recently reported performances. The results indicate that all proposed adsorbents efficiently remove Cr(VI) from the solution, although with varying capacities. The Langmuir adsorption model typically provided better results than the other isotherms. The maximum monolayer adsorption capacities vary from 1.222 mg/g for pristine GO [62] and 1.26 mg/g for MWCNs [45] to 970.87 mg/g for the CNT@Fe_3_N nanocomposite [52]. Most published studies reported that the adsorption of Cr(VI) followed a pseudo-second-order kinetic model, suggesting a chemisorption process. Under acidic conditions, the adsorbent surface becomes positively charged, enhancing the adsorption of anionic chromium species. Multiple removal mechanisms—including electrostatic attraction, ion exchange, surface complexation, and reduction (where electrons are provided by the adsorption site or from an adjacent site), followed by precipitation—have been reported in the literature. These interconnected mechanisms collectively contribute to the efficient removal of Cr(VI) from contaminated aqueous solutions.

It should be noted that nanomaterials were also proposed for the removal of Cr(III) species, although much less often than for Cr(VI) [18,91,92,93,94,95]. Ahmet et al. studied the removal of Cr(III) from a real effluent sample with a concentration of 3477.5 mg/L [91]. At a 1.0 g/100 mL GO dose and 20 min of treatment, the efficiency of this process was 51.88%. Cr(III) adsorption on the graphene oxide surface was attributed mainly to electrostatic interaction as the maximum adsorption was obtained at pH 4.0. The maximum Cr(III) adsorption capacity of 17 mg/g at pH 6.5 was reported for magnetic NiFe_2_O_4_@GO nanocomposite [92]. However, Cr(III) ion removal decreased from 91% to 43% after five sorption/desorption cycles. Bai et al. proposed a GO@alginate hydrogel membrane as an adsorbent with a high adsorption capacity of 118.6 mg/g [94].

## 3. Speciation of Chromium

As chromium toxicity greatly depends on its oxidation state, quantifying all its species is essential to understanding its potential environmental impact and health risk [95,96,97]. The non-chromatographic speciation procedures for chromium using sorptive nanomaterials are generally based on the selective separation and preconcentration of the Cr(III) [98,99,100,101,102] or Cr(VI) [103,104,105,106] forms. The total chromium content is later determined after Cr(III) oxidation or by Cr(VI) reduction. The difference can be used to calculate the concentration of the second species. These procedures can be conducted in batch systems or using a flow-injection methodology [21,30]. Besides the selective separation of one of the forms of chromium, an improvement in the sensitivity of the quantification of the adsorbed species can be obtained. Examples of recent procedures for chromium speciation using nanomaterials are presented in Table 2. The high adsorption capacity of the adsorbent used permits a large sample volume to be uploaded and consequently achieves a good preconcentration factor (EF) value. However, it leads to a decrease in the sampling rate.

Sharma et al. proposed the application of MWCNTs chemically modified with tyrosine or with 4-aminosalicylic acid, as both are selective for Cr(III) adsorption [98]. Chelate formation through various atoms of the functional groups on the adsorbent’s surface was the main mechanism for the uptake of Cr(III). Carbon nanotubes with tyrosine gave a lower detection limit for GFAAS detection, while modification with aminosalicylic acid was characterized by higher values of sampling frequency and preconcentration factor. Magnetic nanoparticles coated with polyaniline (Fe_3_O_4_@cPANI) were also used for the selective separation and preconcentration of Cr(III) at a pH of 8 with an EF equal to 80 [99]. After reduction using hydroxylamine solution, both Cr forms were adsorbed by the same Fe_3_O_4_@PANI nanomaterial at pH 1.0 to determine the total chromium. The very strong affinity of Cr(III) for polythiophene results in a fast adsorption time (1 min) in comparison to the desorption process (15 min) [23]. As can be seen from Table 2, the addition of aniline to the nanocomposite for the synthesis of Fe_3_O_4_@coPANI-PTh for Cr(III) resulted in a higher EF value [100].

**Figure 7:**
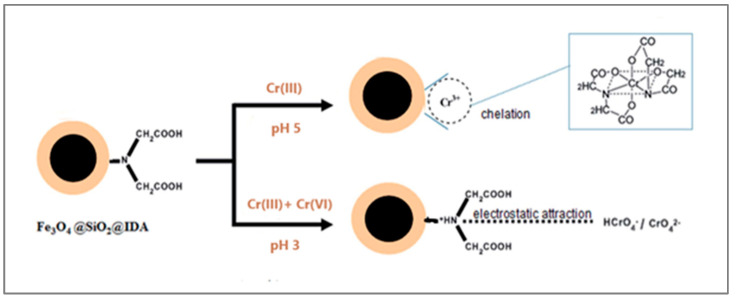
Schematic procedure for the adsorption of Cr(III) and Cr(VI) on Fe_3_O_4_@SiO_2_@IDA nanoparticles for chromium speciation. Reprinted under the terms of a CC BY license from reference [101]. Copyright 2017 Royal Society of Chemistry.

Wei et al. reported a simple and selective method for chromium speciation analysis using magnetic nanoparticles functionalized with iminodiacetic acid (Fe_3_O_4_@SiO_2_@IDA) without additional reduction/oxidation reactions [101]. Quantitative adsorption was obtained in the pH range of 2.5–7 for Cr(III) and 2.5–3.5 for Cr(VI). Under an acidic medium, deprotonated imino and carboxyl groups on the nanosorbent could chelate only Cr(III) ions (Figure 7). The second subsample was adjusted to pH 3 and sonicated with 10 mg of Fe_3_O_4_@SiO_2_@IDA for 10 min. After magnetic separation and elution with the nitric acid solution, the concentration of Cr(VI) was determined. This method exhibited linearity in the range of 20–800 ng/L with limits of detection of 9.1 and 12.8 ng/L for Cr(III) and total Cr, respectively.

The formation of stable complexes of Cr(III) and Cr(VI) with ammonium pyrrolidine dithiocarbamate (APDC) has been used for the selective adsorption of hexavalent chromium on the hybrid nanocomposite MWCNTs@CuAl_2_O_4_@SiO_2_ [102]. The Cr(VI)-APDC complex is adsorbed onto the surface of this nanocomposite within the pH range of 4.5–5.5 through electrostatic attraction, ion exchange, and surface complexation. A small portion of HNO_3_ solution (3 mL) in 10% acetone was used for its elution. Complexes of chromium with dithiocarbamates were also applied by Saboorti, which produced magnetic Fe_3_O_4_@SiO_2_@2-(propylamino-ethyl) dithiocarbamate nanoparticles (Fe_3_O_4_@PAEDTC) which were then fixed within the structures of metal–organic frameworks [103]. Cr(III) was adsorbed on (Fe_3_O_4_@PAEDTC) at pH 2 followed by elution using an acidic EDTA solution. The same operating conditions were used to extract and determine the total amount of chromium but at a sample pH of 5. In another work, Cr(VI)-APDC chelates were adsorbed onto a CoFe_2_O_4_@SiO_2_-C_8_ nanocomposite at a pH of 2 with a high recovery rate of 96.8% and both chromium species chelated with APDC at pH 6.0 [104]. The concentration of Cr (III) ions in the solution was calculated by the difference between the total Cr and the Cr (VI) level. An efficient magnetic SPE method was described for the preconcentration of Cr(VI) using an as-prepared NiO-NiFe_2_O_4_@LDH nanocomposite [105]. In contrast to Cr(III), a very high EF value of 250 was obtained for Cr(VI) adsorption at pH 6 ions and elution with hydroxyl ammonium chloride.

Successful examples of chromium speciation in environmental water and soil samples using nanomaterials were reported by Pang et al. [106]. On-line coupling of in-tube solid-phase microextraction with chromatographic analysis was applied for the simultaneous adsorption and preconcentration of both chromium species and their APDC complexes, formed in a sample solution at pH of 5.5 on a monolith-based capillary column modified with Fe_3_O_4_ nanoparticles. Hydrogen bonds and hydrophobic and dipole–dipole interactions played a significant role in the enrichment process. Elution of chromium complexes was carried out using acetonitrile and the eluant was delivered to the HPLC/DAD system for separation and detection. It was shown that the system used displays good stability and can be reused at least 100 times without a loss of extraction efficiency.

The analytical performance of the proposed method for chromium speciation in different kinds of water was confirmed by analyzing samples spiked with Cr(III) and Cr(VI) species. In most cases, recoveries for both ions above 95% were achieved, indicating the reliability of a given procedure. The accuracy of this method should be evaluated using appropriate certificated reference materials (CRMs). However, the contents of the analyzed CRMs only been certified for total chromium [23,100,101,104].

## 4. Conclusions

Nanomaterials continue to attract significant interest for chromium removal, preconcentration, and speciation analysis. They offer a large surface area and excellent thermal and mechanical stability. Hybrid nanomaterials can be engineered by incorporating functionalized groups and various fillers to enhance their adsorption performance. Additionally, magnetization facilitates their separation after adsorption treatment. However, it should be noted that the synthesis of these adsorbents is often time consuming due to the multi-stage processes involved and requires advanced technical expertise.

Under acidic conditions, Cr(VI) anions are adsorbed through electrostatic interactions with the positively charged adsorbent surface and subsequently reduced to the less toxic Cr(III) by electron-donating groups and iron species. Furthermore, the adsorption process can be governed by surface complexation through chemisorption and pore filling. The necessity of maintaining optimal acidic conditions for Cr(VI) removal may pose a challenge for industrial applications on a larger scale. The reduction of Cr(VI) to Cr(III) is particularly important when spent materials are either discarded or regenerated for extended use. The authors of the cited studies demonstrated the potential of reusing their nanomaterials up to five times [67,74,75]. The decline in Cr(VI) removal in the subsequent cycles could be explained by partial damage of the nanomaterial’s structure by the strong acid/alkali treatment. The development of mild reagents for desorbing Cr(III) and the synthesis of nanomaterials with strong chemical stabilities are necessary to promote their reuse. Furthermore, the authors did not address the fate of the Cr(III) solutions produced after the regeneration steps or the precipitated trivalent chromium forms. Although these forms exhibited low solubility and mobility, reducing the risk of groundwater contamination, they remain exposed to environmental conditions that trigger their release and oxidation [107,108].

The long-term stability of adsorbents designed for Cr(VI) removal has rarely been investigated. It would be valuable to assess the stability of synthesized nanocomposites by performing adsorption experiments for a few months after their synthesis. Currently, the procedures described are mostly limited to batch experiments conducted on a laboratory scale.

Adsorbents used in chromium speciation analysis have demonstrated a preferential affinity for a specific chromium oxidation state, such as Cr(III) or Cr(VI). After an oxidation or reduction reaction, the total chromium content is determined from a separate aliquot of the sample, and the concentration of the second species is calculated using the difference. This methodology has several drawbacks, including incomplete conversion, the risk of contamination, and additional time-consuming steps. A preferable approach would be to separate individual species followed by their direct determination, as this requires only minimal sample pretreatment. The detection limits of recently developed procedures for Cr(VI) are suitable for most regulatory requirements across different countries.

In the future, separation and removal procedures that integrate adsorption processes with membrane techniques or fluidized bed systems could be explored. The main challenge lies in applying these methods to large-scale wastewater treatment while maintaining high removal efficiency. Polymeric hybrid aerogel membranes, which are nanostructured materials, are gaining popularity in various applications due to their ability to be modified with different nanomaterials and nanofillers. Kong et al. have already synthesized a cross-linked graphene oxide@chitosan composite aerogel, which exhibited excellent adsorption properties for Cr(VI) removal [75].

## Figures and Tables

**Table 1:** Examples of the recent application of nanomaterials for Cr(VI) removal.

Nanomaterial	S_BET_ (m^2^/g)	Sample pH	Contact Time	q_max_ (mg/g)		Ref.
CNTs	–	2.5	60 min	SWCNTs	2.35	[45]
				MWCNTs	1.26	
CNTs@CaO_2_	–	3	2 h	81% of removal		[48]
MWCNTs@FeMnO_4_	360	2	24 h	47.25		[49]
CNT@Fe@N	158.71	2	6 h	27.47		[51]
CNTs@Fe_3_N	116.4	1	10 min	970.87		[52]
MWNTs modified with orange juice	21.66	2	3 h	60.91		[57]
GO	–	3	2 h	41.27		[61]
GO	–	4	40 min	1.222		[62]
GO@Fe_3_O_4_	194.6	2	60 min	140.8		[64]
GO@SiO_2_	_	3	24 h	92.3% removal		[66]
PAH@AS@GO	261.6	2	20 min	373.1		[67]
PAH@AS@GO@Fe_3_O_4_		2	20 min	219		
GO@ZnO	32.95	8.02	720 min	3.69		[68]
GO@zinc molybdate	121.18	2	120 min	20.40		[69]
GO@AHMT		3	100 min	734.2		[72]
GO@CS	–	2	420 min	104.16		[74]
GO@Fe_3_O_4_@CS	5.4	2	40 min	100.51		[75]
GO@FeO(OH)@CS	–	3	64 h	63.19		[77]
rGO@Fe-Ni	119.08	5	20 min	197.43		[78]
rGO@ZnO	29.418	1.5	24 h	13.52		[79]
GO@NiFe@LDH	145	6–7	–	53.6		[82]
GO@MoS_2_	4.58	2	200 min	43.95		[86]
rGO@MoS_2_	79.39	2	150 min	62.10		[87]
CS@TiO_2_	26	2	30 min	488		[88]
CS@SiO_2_@TiO_2_	–	2	120 min	182.43		[89]
CoFe_2_O_4_@SiO_2_-NH_2_	128.48	2		126.8		[90]

S_BET_—surface area by the BET equation; SWCNTs—single-walled carbon nanotubes; MWCNTs—multi-walled carbon nanotubes; MWCNT@FeMnO_4—_carbon nanotubes modified by iron-manganese binary oxide; CNT@Fe@N—N-doped magnetic carbon nanotubes; rGO@Fe-Ni—reduced graphene oxide decorated with Fe and Ni nanoparticles; PAH@AS@GO—poly(allylamine hydrochloride) cross-linked amino silane-modified graphene oxide; CS—chitosan; LDH—layered double hydroxide.

**Table 2:** Examples of recent procedures for chromium speciation using nanomaterials.

Nanomaterial	Conditions		Eluent	EF	Ref.
	pH	Time (min)			
Selective sorption of Cr(III)					
MWCNTs-TYR	4	120	0.2 M HNO_3_	93	[98]
MWCNTs-ASA	6	100	0.3 M HNO_3_	97	
Fe_3_O_4_@PTh	7	1	3 M HCl	27	[23]
Fe_3_O_4_@PANI	8	5	0.2% TU + 2 M HCl	80	[99]
Fe_3_O_4_@PANI-PTh	10	5	0.2% TU + 1 M HNO_3_	38.5	[100]
Fe_3_O_4_@SiO_2_@IDA	3.6	10	1.5 M HNO_3_	100	[101]
Selective sorption of Cr(VI)					
MWCNTs@CuAl_2_O_4_@SiO_2_	5		3 M HNO_3_ in 10% acetone	17	[102]
MOF-Fe@PAEDTC	2	6.5	0.6 M EDTA + 0.3 M HNO_3_	17.6	[103]
CoFe_2_O_4_@SiO_2_-C_8_ + APDC	6	10	Ethanol	200	[104]
NiO/NiFe_2_O_4_/LDH	6	15	10% [NH_4_OH]Cl	250	[105]

EF—enrichment factor (ratio of the sample volume to eluent volume); TYR—tyrosine; ASA—aminosalicylic acid; IDA—iminodiacetic acid; PTh—polythiophene; PANI—polyaniline; APDC—ammonium pyrrolidine dithiocarbamate; PAEDTC—2-(propylamino-ethyl) dithiocarbamate; MOF—metal–organic framework; LDH—layered double hydroxide.

## Data Availability

No new data were created or analyzed in this study. Data sharing is not applicable to this article.

## References

[1] Tumolo M., Ancona V., De Paola D., Losacco D., Campanale C., Massarelli C., Uricchio V.F. (2020). Chromium Pollution in European Water, Sources, Health Risk, and Remediation Strategies: An Overview. Int. J. Environ. Res. Public Health.

[2] Lewicki S., Zdanowski R., Krzyżowska M., Lewicka A., Dębski B., Niemcewicz M., Goniewicz M. (2024). The role of Chromium (III) in the organism and its possible use in diabetes and obesity treatment. Ann. Agric. Environ. Med..

[3] DesMarais T.L., Costa M. (2019). Mechanisms of Chromium-Induced Toxicity. Curr. Opin. Toxicol..

[4] Ray A., Jankar J.S. (2022). A Comparative Study of Chromium: Therapeutic Uses and Toxicological Effects on Human Health. J. Pharmacol. Pharmacother..

[5] Monga A., Fulke A.B., Dasgupta D. (2022). Recent developments in essentiality of trivalent chromium and toxicity of hexavalent chromium: Implications on human health and remediation strategies. J. Hazard. Mater. Adv..

[6] Vaiopoulou E., Gikas P. (2020). Regulations for chromium emissions to the aquatic environment in Europe and elsewhere. Chemosphere.

[7] World Health Organization (2020). Chromium in Drinking Water. Background Document for Development of WHO Guidelines for Drinking-Water Quality.

[8] Procópio V.A., Pereira R.M., Lange C.N., Freire B.M., Batista B.L. (2023). Chromium Speciation by HPLC-DAD/ICP-MS: Simultaneous Hyphenation of Analytical Techniques for Studies of Biomolecules. Int. J. Environ. Res. Public Health.

[9] Pechancová R., Gallo J., Milde D., Pluháček T. (2020). Ion-exchange HPLC-ICP-MS: A new window to chromium speciation in biological tissues. Talanta.

[10] Saraiva M., Chekri R., Leufroy A., Guérin T., Sloth J.J., Jitaru P. (2021). Development and validation of a single run method based on species-specific isotope dilution and HPLC-ICP-MS for simultaneous species interconversion correction and speciation analysis of Cr(III)/Cr(VI) in meat and dairy products. Talanta.

[11] Badawy M.E.I., El-Nouby M.A.M., Kimani P.K., Lim L.W., Rabea E.I. (2020). A review of the modern principles and applications of solid-phase extraction techniques in chromatographic analysis. Anal. Sci..

[12] Matczuk M., Ruzik L., Kepplen B.K., Timerbaew A.T. (2023). Nanoscale Ion-Exchange Materials: From Analytical Chemistry to Industrial and Biomedical Applications. Molecules.

[13] Moral A., Borull F., Moarcé R.M., Fontanales N. (2024). Novel materials for sorptive extraction techniques for the analysis of environmental water samples. TrAC Trends Anal. Chem..

[14] Corps Ricardo A.I., Abujaber F., Guzmán Bernardo F.J., Rodríguez Martín-Doimeadios R.C., Ríos Á. (2020). Magnetic solid phase extraction as a valuable tool for elemental speciation analysis. Trends Environ. Anal. Chem..

[15] Filik H., Avan A.A. (2019). Magnetic nanostructures for preconcentration, speciation and determination of chromium ions: A review. Talanta.

[16] Liu B., Xin Y.N., Zou J., Khoso F.M., Liu Y.P., Jiang X.Y., Peng S., Yu J.G. (2023). Removal of Chromium Species by Adsorption: Fundamental Principles, Newly Developed Adsorbents and Future Perspectives. Molecules.

[17] Erbas Z., Ozalp O., Matin A.A., Soylak M. (2024). Speciation of Chromium by Magnetic Solid Phase Microextraction Using an Activated Charcoal-Molybdenum (IV) Selenide-Magnetite Composite with Flame Atomic Absorption Spectrometric (FAAS) Detection. Anal. Lett..

[18] Almeida J.C., Cardodo C.E.D., Taavares D.S., Freitas R., Trindadw T., Vale C., Pereira E. (2019). Chromium removal from contaminated waters using nanomaterials—A review. TrAC Trends Anal. Chem..

[19] Pyrzynska K. (2020). Nanomaterials in speciation analysis of metals and metalloids. Talanta.

[20] Arain M.B., Ali I., Yilmaz E., Soylak M. (2018). Nanomaterial’s based chromium speciation in environmental samples: A review. TrAC Trends Anal. Chem..

[21] Posta J., Nagy D., Kapitány S., Beni Ă. (2019). A comparison study of analytical performance of chromium speciation methods. Microchem. J..

[22] López-García I., Marín-Hernández J.J., Hernández-Córdoba M. (2020). Speciation of chromium in waters using dispersive micro-solid phase extraction with magnetic ferrite and graphite furnace atomic absorption spectrometry. Sci. Rep..

[23] Sodan N.E., Elci Ş.G., Kartal A.A., Hol A., Elci L. (2021). Speciation and preconcentration of chromium in real samples by magnetic polythiophene nanoparticle solid-phase extraction (SPE) coupled with microsampling injection—Flame atomic absorption spectrometry (FAAS). Instrum. Sci. Technol..

[24] Çiçek Özkul S.L., Kaba I., Ozdemir Olgun F.A. (2024). Unravelling the potential of magnetic nanoparticles: A comprehensive review of design and applications in analytical chemistry. Anal. Meth..

[25] Sajid M., Płotka-Wasylka J. (2020). Nanoparticles: Synthesis, characteristics, and applications in analytical and other sciences. Microchem. J..

[26] Sheoran K., Kaur H., Siwal S.S., Saini A.K., Dai-Viet V., Thakur V.K. (2022). Recent advances of carbon-based nanomaterials (CBNMs) for wastewater treatment: Synthesis and application. Chemosphere.

[27] Kaur H., Devi N., Siwal S.S., Alsanie W.F., Thakur M.K., Thaku V.K. (2023). Metal-Organic Framework-Based Materials for Wastewater Treatment: Superior Adsorbent Materials for the Removal of Hazardous Pollutants. ACS Omega.

[28] Lin Y., Tian Y., Sun H., Hagio T. (2021). Progress in modification of 3D graphene-based adsorbents for environmental applications. Chemosphere.

[29] Kaymaz S.V., Nobar H.M., Sarıgül H., Soylukan C., Akyüz L., Yüce M. (2023). Nanomaterial surface modification toolkit: Principles, components, recipes, and applications. Adv. Colloid Interface Sci..

[30] Murali Manoj G., Shalini M., Thenmozhi K., Kumar Ponnusamy V., Hari S. (2024). Recent advancements in the surface modification and functionalization of magnetic nanomaterials. Appl. Surf. Sci. Adv..

[31] Li H., Chen X., Shen D., Wu F., Pleixats R., Pan J. (2021). Functionalized silica nanoparticles: Classification, synthetic approaches and recent advances in adsorption applications. Nanoscale.

[32] Woźniak J., Nawała J., Dziedzic D., Popiel S. (2024). Overview of Liquid Sample Preparation Techniques for Analysis, Using Metal-Organic Frameworks as Sorbents. Molecules.

[33] Trzonkowska L., Leśniewska B., Godlewska-Żyłkiewicz B. (2016). Recent Advances in On-Line Methods Based on Extraction for Speciation Analysis of Chromium in Environmental Matrices. Crit. Rev. Anal. Chem..

[34] Yuan X., Li J., Luo L., Zhong Z., Xie X. (2023). Advances in Sorptive Removal of Hexavalent Chromium (Cr(VI)) in Aqueous Solutions using Polymeric Materials. Polymers.

[35] Herrero-Latorre C., Barcielo-García J., García-Martín S., Pena-Crescente R.M. (2018). Graphene and carbon nanotubes as solid phase extraction sorbents for the speciation of chromium: A review. Anal. Chim. Acta.

[36] García A., Rodríguez B., Rosales M., Quintero Y.M., GSaiz P., Reizabal A., Wuttke S., Celaya-Azcoaga L., Valverde A., Fernández de Luis R. (2022). State-of-the-Art of Metal-Organic Frameworks for Chromium Photoreduction vs. Photocatalytic Water Remediation. Nanomaterials.

[37] Nemati S.S., Dehghan G., Khataee A., Alidokht L., Kudaibergenov N. (2024). Layered double hydroxides as versatile materials for detoxification of hexavalent chromium: Mechanism, kinetics, and environmental factors. J. Environ. Chem. Eng..

[38] Staszak K., Kruszelnicka I., Ginter-Kramarczyk D., Góra W., Baraniak M., Regel-Rosocka M. (2023). Advances in the removal of Cr(III) from Spent Industrial Effluents—A Review. Materials.

[39] Gahrouei A.E., Rezapour A., Pirooz M., Pourebrahini S. (2024). From classic to cutting-edge solutions: A comprehensive review of materials and methods for heavy metal removal from water environments. Desalin. Water Treat..

[40] Xu H., Zhang H., Qin C., Li X., Xu D., Zhao Y. (2024). Groundwater Cr(VI) contamination and remediation: A review from 1999 to 2022. Chemosphere.

[41] Maitlo H.A., Kim K.H., Kumar V., Kim S., Park J.W. (2019). Nanomaterials-based treatment options for chromium in aqueous environments. Environ. Inter..

[42] Assefa H., Singh S., Olu F.E., Dhanjal D.S., Mani D., Khan N.A., Singh J., Praveen C., Ramamurthy P.C. (2024). Advances in adsorption technologies for hexavalent chromium removal: Mechanisms, materials, and optimization strategies. Desalin. Water Treat..

[43] Wang J., Guo X. (2023). Adsorption kinetics and isotherm models of heavy metals by various adsorbents: An overview. Crit. Rev. Environ. Sci. Technol..

[44] Mohammadmoradi P., Taheri S., Bryant S.L., Apostolos Kantzas A. (2018). Solvent diffusion and dispersion in partially saturated porous media: An experimental and numerical pore-level study. Chem. Eng. Sci..

[45] Dehghani M.H., Taher M.M., Bajpai A.K., Heibati B., Tyagi I., Asif M., Agarwal S., Gupta V.K. (2015). Removal of noxious Cr(VI) ions using single-walled carbon nanotubes and multi-walled carbon nanotubes. Chem. Eng. J..

[46] Mpouras T., Polydera A., Dermatas D., Verdone N., Vilardi G. (2021). Multiwall carbon nanotubes application for treatment of Cr(VI)-contaminated groundwater; Modeling of batch & column experiments. Chemosphere.

[47] Huang Y., Song K., Luo W., Yang J. (2020). Adsorption and reduction of Cr(VI) by hydroxylated multiwalled carbon nanotubes: Effect of humic acid and surfactants. Environ. Sci. Pollut. Res..

[48] Chen Z., Fu D., Koh K.Y., Chen J.P. (2022). A new carbon nanotube modified by nano CaO_2_ for removal of chromate and phosphate from aqueous solutions. Chem. Eng. J..

[49] Ma C., Liu D., Deng S., Vakili M. (2024). Efficient chromium (VI) removal with zirconium oxide-carbon nanotubes via filtration-steam hydrolysis. Chem. Eng. Res. Des..

[50] Ma T., Wu Y., Liu N., Yan C. (2022). Adsorption behavior of Cr(VI) and As(III) on multiwall carbon nanotubes modified by iron-manganese binary oxide (FeMnOx/MWCNTs) from aqueous solution. Sep. Sci. Technol..

[51] Cheng S., Zeng X., Liu P. (2024). One-step synthesis of magnetic N-doped carbon nanotubes derived from waste plastics for effective Cr(VI) removal. Arab. J. Chem..

[52] Huang J., Cao Y., Qin B., Zhong G., Zhang J., Yu H., Wang H., Peng F. (2019). Highly efficient and acid-corrosion resistant nitrogen doped magnetic carbon nanotubes for the hexavalent chromium removal with subsequent reutilization. Chem. Eng. J..

[53] Liu K., Zhao D., Hu Z., Xiao Y., He C., Jiang F., Zhao N., Zhao C., Zhang W., Qiu R. (2023). The adsorption and reduction of anionic Cr(VI) in groundwater by novel iron carbide loaded on N-doped carbon nanotubes: Effects of Fe-confinement. Chem. Eng. J..

[54] Fenta E.W., Mebratie B.A. (2024). Advancements in carbon nanotube-polymer composites: Enhancing properties and applications through advanced manufacturing techniques. Heliyon.

[55] Mahpishanian S., Ahmadian-Alam L., Foudazi R. (2023). Porous polymer nanocomposites containing single-walled carbon nanotubes for chromium (VI) removal from water. React. Funct. Polym..

[56] Jia D., Jing Z., Duan Y., Li J. (2022). Ultrafast removal of Cr(VI) ions using polyamine modified carbon nanotubes. J. Taiwan Inst. Chem. Eng..

[57] Amaku J.F., Taziwa R. (2024). Aqueous removal of Cr(VI) by *Citrus sinensis* juice-coated multi-walled carbon nanotubes. Chem. Pap..

[58] Pena-Pereira F., Romero F., de la Calle I., Lavilla I., Bendicho C. (2021). Graphene-based nanocomposites in analytical extraction processes. TrAC Trends Anal. Chem..

[59] Nanjundappa V.S., Ramakrishnappa T., Prakash H.R., Praveen B.M. (2023). Efficient strategies to produce Graphene and functionalized graphene materials: A review. Appl. Surf. Sci. Adv..

[60] Tarcan R., Todor-Boer O., Petrovai I., Leorean C., Astilean S., Botiz I. (2020). Reduced graphene oxide today. J. Mater. Chem. C.

[61] Khdoor Z., Makharza S., Qurie M., Fohely F., Taha S.A. (2024). Hampe, Removal of toxic hexavalent chromium via graphene oxide nanoparticles: Study of kinetics, isotherms, and thermodynamics. RSC Adv..

[62] Mondal N.K., Chakraborty S. (2020). Adsorption of Cr(VI) from aqueous solution on graphene oxide (GO) prepared from graphite: Equilibrium, kinetic and thermodynamic studies. Appl. Water Sci..

[63] Guo T., Bulin C. (2024). Adsorption-reduction behavior of hexavalent chromium on Fe_3_O_4_-graphene oxide surface with special implication on environmental remediation. Ceram. Int..

[64] Mahvi A.H., Balarak D., Bazrafshan E. (2021). Remarkable reusability of magnetic Fe_3_O_4_-graphene oxide composite: A highly effective adsorbent for Cr(VI) ions. Int. J. Environ. Anal. Chem..

[65] Zhang F., Song S., Wang H., Wang W., Yang Y., Li Y. (2020). Efficient removal of hexavalent chromium and Congo red by graphene oxide/silica nanosheets with multistage pores. J. Chem. Eng. Data.

[66] Jibin K.P., Augustine S., Velayudhan P., George S.K., Poulose S.V., Thomas S. (2023). Unleashing the Power of Graphene-Based Nanomaterials for Chromium(VI) Ion Elimination from Water. Crystals.

[67] Bao S., Wang Y., Wei Z., Yang W., Yu Y., Sun Y. (2021). Amino-assisted AHMT anchored on graphene oxide as high performance adsorbent for efficient removal of Cr(VI) and Hg(II) from aqueous solutions under wide pH range. J. Hazard. Mater..

[68] Singh S., Anil A.G., Khasnabis S., Kumar V., Nath B., Adiga V., Kumar Naik T.S., Subramanian S., Kumar V., Singh J. (2022). Sustainable removal of Cr(VI) using graphene oxide-zinc oxide nanohybrid: Adsorption kinetics, isotherms and thermodynamics. Environ. Res..

[69] Merija K.S., Rahulan K.M., Sujatha R.A., Little Flower N.A. (2023). Adsorption of hexavalent chromium from water using graphene oxide/zinc molybdate nanocomposite: Study of kinetics and adsorption isotherms. Front. Energy Res..

[70] Bao S., Yang W., Wang Y., Yu Y., Sun Y. (2021). Highly efficient and ultrafast removal of Cr(VI) in aqueous solution to ppb level by poly(allylamine hydrochloride) covalently cross-linked amino-modified graphene oxide. J. Hazard. Mater..

[71] Wu B., Hong M., Wu Q., Li X., Zhao Y., Wang S., Wang Z. (2025). New Schiff base covalently bonded graphene oxide for removing chromium(VI) from surface runoff. Environ. Res..

[72] Kaczorowska M.A., Bożejewicz D. (2024). The Application of Chitosan-Based Adsorbents for the Removal of Hazardous Pollutants from Aqueous Solutions—A Review. Sustainability.

[73] Matei E., Predescu A.M., Râpă M., Tarcea C., Pantilimon C.M., Favier L., Berbecaru A.C., Sohaciu M., Predescu C. (2019). Removal of Chromium(VI) from Aqueous Solution Using a Novel Green Magnetic Nanoparticle–Chitosan Adsorbent. Anal. Lett..

[74] Samuel M.S., Bhattacharya J., Raj S., Santhanam N., Singh H., Singh N.D.P. (2019). Efficient removal of Chromium(VI) from aqueous solution using chitosan grafted graphene oxide (CS-GO) nanocomposite. Int. J. Biol. Macromol..

[75] Kong D., He L., Li H., Zhang F., Song Z. (2021). Preparation and characterization of graphene oxide/chitosan composite aerogel with high adsorption performance for Cr(VI) by a new crosslinking route. Colloids Surf. A Physicochem. Eng. Asp..

[76] Subedi N., Lähde A., Abu-Danso E., Iqbal J., Bhatnagar A. (2019). A comparative study of magnetic chitosan (Chi@Fe_3_O_4_) and graphene oxide modified magnetic chitosan (Chi@Fe_3_O_4_GO) nanocomposites for efficient removal of Cr(VI) from water. Int. J. Biol. Macromol..

[77] Liu Y., Shan H., Zeng C., Zhan H., Pang Y. (2022). Removal of Cr(VI) from Wastewater Using Graphene Oxide Chitosan Microspheres Modified with α-FeO(OH). Materials.

[78] Kang Z., Gao H., Hu Z., Jia X., Wen D. (2022). Ni-Fe/Reduced Graphene Oxide Nanocomposites for Hexavalent Chromium Reduction in an Aqueous Environment. ACS Omega.

[79] Philip R.S., Aparna N., Meril M. (2024). Hexavalent chromium removal using reduced graphene oxide-zinc oxide composite fabricated via simple pyrolysis method. App. Surf. Sci. Adv..

[80] da Gama B.M.V., Selvasembian R., Giannakoudakis D.A., Triantafyllidis K.S., McKay G., Meili L. (2022). Layered double hydroxides as rising-star adsorbents for water purification: A brief discussion. Molecules.

[81] Wani A.A., Khan A.M., Yahiya G., Manea K., Shahadat M., Ahammad S.Z., Ali S.W. (2020). Graphene-supported organic-inorganic layered double hydroxides and their environmental applications: A review. J. Clean. Prod..

[82] Zheng Y., Cheng B., You W., Yu J., Ho W. (2019). 3D hierarchical graphene oxide-NiFeLDH composite with enhanced adsorption affinity to Congo red, methyl orange and Cr(VI) ions. J. Hazard. Mater..

[83] Lv X., Qin X., Wang K., Peng Y., Wang P., Jiang G. (2019). Nanoscale zero valent iron supported on MgAl-LDH-decorated reduced graphene oxide: Enhanced performance in Cr(VI) removal, mechanism and regeneration. J. Hazard. Mater..

[84] Jiang R., Xiao M., Zhu H.Y., Zang X., Zhao D.X., Zhu J.Q., Long Y.K., Wang Q. (2024). Intriguing and boosting molybdenum sulfide (MoS_2_)-based materials for decontamination and purification of wastewater/seawater: An upgraded review. Sep. Purif. Technol..

[85] Santra S., Ali M.S., Karmakar S., Chattopadhyay D. (2024). Molybdenum disulfide: A nanomaterial that is paving the way toward a sustainable future. Mater. Today Sustain..

[86] Han Q., Yu J., Poon S., Sun L., Teli M., Liu B., Chen H., Wang K., Wang Z., Mi B. (2022). Highly efficient removal and sequestration of Cr(VI) in confined MoS_2_ interlayer Nanochannels: Performance and mechanism. Sep. Purif. Technol..

[87] Zhou S., Gao J., Wang S., Fan H., Huang J., Liu Y. (2020). Highly efficient removal of Cr(VI) from water based on graphene oxide incorporated flower-like MoS_2_ nanocomposite prepared in situ hydrothermal synthesis. Environ. Sci. Pollut. Res..

[88] Al-Obaidi N.S., Sadeq Z.E., Mahmoud Z.H., Najem Abd A., Al-Mahdawi A.S., Ali F.K. (2023). Synthesis of Chitosan-TiO_2_ Nanocomposite for Efficient Cr(VI) Removal from Contaminated Wastewater Sorption Kinetics, Thermodynamics and Mechanism. J. Oleo Sci..

[89] El Kaim Billah R., Shekhawat A., Mansouri S., Majdoubi H., Agunaou M., Soufiane A., Jugade R. (2022). Adsorptive removal of Cr(VI) by Chitosan-SiO_2_-TiO_2_ nanocomposite. Environ. Nanotechnol. Monit. Manag..

[90] Zhou D., Wang J., Chen H., Ge X., Wang X. (2022). Enhanced Cr(VI) removal by hierarchical CoFe_2_O_4_@SiO_2_-NH_2_ via reduction and adsorption processes. New J. Chem..

[91] Ahmed S., Fatema Tuj Z., Mahdi M.M., Mahmudunnabi D.M., Choudhury T.R., Alam M.Z., Nurnabi M. (2021). Synthesis and characterization of graphene oxide for removal of Cr(III) from tannery effluent. Desalin. Water Treat..

[92] Ortiz-Quiñonez J.L., Cancino-Gordillo F.E., Pal U. (2023). Removal of Cr(III) Ions from Water Using Magnetically Separable Graphene-Oxide-Decorated Nickel Ferrite Nanoparticles. ACS Appl. Nano Mater..

[93] Zhou R., Yang S., Tao E., Xiao X., Liu L., Li Y. (2021). Mild-method synthesized GO-TiO_2_ retains oxygen-containing functional groups as an effective adsorbent. J. Solid State Chem..

[94] Bai C., Wang L., Zhu Z. (2022). Adsorption of Cr(III) and Pb(II) by graphene oxide/alginate hydrogel membrane: Characterization, adsorption kinetics, isotherm and thermodynamics studies. Inter. J. Biol. Macromol..

[95] Prasad S., Yadav K.K., Kumar S., Gupta N., Cabral-Pinto N.M.S., Rezania S., Radwan N., Alam J. (2021). Chromium contamination and effect on environmental health and its remediation: A sustainable approaches. J. Environ. Manag..

[96] Pechancová R., Pluháček T., Milde D. (2019). Recent advances in chromium speciation in biological samples. Spectrochim. Acta Part B.

[97] Zulfiqar U., Haider F.U., Ahmad M., Hussain S., Maqsood M.F., Ishfaq M., Shahzad B., Waqas M.M., Ali B., Nayyab T.M. (2023). Chromium toxicity, speciation, and remediation strategies in soil-plant interface: A critical review. Front. Plant Sci..

[98] Sharma N., Tiwari S., Saxena R. (2020). Comparative Insight into the Performance of Two Different Amine-Functionalized CNTs for the Chemical Speciation of Chromium. Chem. Sel..

[99] Çaylak O. (2024). Chromium speciation in water using magnetic polyaniline nanoparticles coupled with microsampling injection-flame atomic absorption spectroscopy. Turk. J. Chem..

[100] Elçi Ş.G. (2020). Speciation of chromium in beverages and seasoning samples by magnetic solid-phase extraction and microsample injection system flame atomic absorption spectrometry. Cumhur. Sci. J..

[101] Wei W., Zhao B., He M., Chen B., Hu B. (2017). Iminodiacetic acid functionalized magnetic nanoparticles for speciation of Cr(III) and Cr(VI) followed by graphite furnace atomic absorption spectrometry detection. RSC Adv..

[102] Hassan Ahmed H.E., Soylak M. (2024). A MWCNTs@CuAl_2_O_4_@SiO_2_ Nanocomposite for the Speciation of Cr(III), Cr(VI), and Total Chromium Prior to High-Resolution Continuum Source Flame Atomic Absorption Spectrometric Determination. Water Air Soil Pollut..

[103] Saboori A. (2017). A nanoparticle sorbent composed of MIL-101(Fe) and dithiocarbamate-modified magnetite nanoparticles for speciation of Cr(III) and Cr(VI) prior to their determination by electrothermal AAS. Microchim. Acta.

[104] Ghiasi A., Malekpour A. (2020). Octyl coated cobalt-ferrite/silica core-shell nanoparticles for ultrasonic assisted-magnetic solid-phase extraction and speciation of trace amount of chromium in water samples. Microchem. J..

[105] Abdolmohammad-Zadeh H., Ayazi Z., Veladi M. (2021). Nickel oxide/nickel ferrite/layered double hydroxide nanocomposite as a novel magnetic adsorbent for chromium speciation. Microchem. J..

[106] Pang J., Chen H., Huang X. (2021). Magnetism-assisted in-tube solid-phase microextraction for the on-line chromium speciation in environmental water and soil samples. Microchem. J..

[107] Lei D., Zhang Y., Sun X., Li L., Zhao Q., Peng X., Xue J., Wang Y., Zhang J. (2025). Sulfite-Induced Release and Oxidation of Cr(III) in Reduced Chromite Ore Processing Residue under Visible Light: The Critical Role of Fe(IV) Intermediates. Environ. Sci. Technol..

[108] Li Y., Wang Y., Huang X., Zhou W., Liang J., Liu Y., Shen Y., Tong M. (2025). The effect of phosphate on the stability of Cr(OH)_3_ and Cr_x_Fe_1−x_(OH)_3_ in the presence of MnO_2_: Competition between dissolution, adsorption and oxidation. J. Hazard. Mater..

